# Caregiver Neurotic Personality Trait and Elder Abuse—The Moderating Role of Change in the Level of Caregiver Burden

**DOI:** 10.1093/geroni/igaa057.1243

**Published:** 2020-12-16

**Authors:** Boye Fang, Elsie Yan, Yaolin Pei, Xiaozhao Yang

**Affiliations:** 1 Sun Yat-Sen University, Guangzhou, China; 2 The Hong Kong Polytechnic University, Hong Kong, China; 3 New York University, New York, New York, United States; 4 Sun Yat-Sen University, Guangzhou, United States

## Abstract

Objectives: To investigate whether caregiver neuroticism has an effect on subsequent occurrence of elder abuse and whether change in the level of caregiver perceived burden alters this relationship. Methods: Using two-year longitudinal data, we analyzed a consecutive sample of 800 Chinese family caregivers and their care recipients with dementia recruited from the geriatric and neurological departments of three Grade-A hospitals in People’s Republic of China (PRC). All the participatory dyads were assessed between September 2015 and February 2016 and followed for two years. Results: Significant increase in the prevalence was found for physical and psychological abuse, caregiver neglect, and financial exploitation. Caregivers high in neuroticism were more likely to engage in subsequent physical and psychological abuse, however, change in the level of caregiver perceived burden altered this association. Specifically, absence and alleviation of care burden during the two-year observation prevented the subsequent occurrence of physical and psychological abuse. Although caregiver neuroticism was also associated with subsequent caregiver neglect, caregiver perceived burden did not appear to have an impact on this relationship. Discussion: This study provided evidence that caregiver neuroticism was associated with subsequent physical and psychological abuse, while change in the level of caregiver perceived burden may alter this trajectory. These findings suggest the importance of implementing caregiver-centered intervention and prevention programs for elder abuse by specifically targeting at caregivers’ behaviors related to their neurotic personality trait and cognitive appraisal of caregiving stressors associated with such personality trait.

